# The prokaryotic V4R domain is the likely ancestor of a key component of the eukaryotic vesicle transport system

**DOI:** 10.1186/1745-6150-3-2

**Published:** 2008-01-25

**Authors:** Mircea Podar, Mark A Wall, Kira S Makarova, Eugene V Koonin

**Affiliations:** 1 Biosciences Division and the Bioenergy Science Center, Oak Ridge National Laboratory, 1 Bethel Valley Rd, Oak Ridge, TN 37831, USA; 2 Bioinformatics Department, Verenium Corporation, 4955 Directors Place, San Diego, CA 92121, USA; 3 National Center for Biotechnology Information, National Library of Medicine, National Institutes of Health, Bethesda, MD 20894, USA

## Abstract

Intracellular vesicle traffic that enables delivery of proteins between the endoplasmic reticulum, Golgi and various endosomal subcompartments is one of the hallmarks of the eukaryotic cell. Its evolutionary history is not well understood but the process itself and the core vesicle traffic machinery are believed to be ancient. We show here that the 4-vinyl reductase (V4R) protein domain present in bacteria and archaea is homologous to the Bet3 subunit of the TRAPP1 vesicle-tethering complex that is conserved in all eukaryotes. This suggests, for the first time, a prokaryotic origin for one of the key eukaryotic trafficking proteins.

This article was reviewed by Gaspar Jekely and Mark A. Ragan

## Findings

The specificity of eukaryotic vesicle traffic is controlled by dozens of proteins and protein complexes that ensure correct docking, promote membrane fusion between vesicles, and target distinct compartments. Evolutionary analyses using genomic data from a wide range of eukaryotes indicate that the trafficking system is ancient and that most if not all the core constituents were present in the last eukaryotic common ancestor (LECA)[1]. However, no direct relationship has been so far detected between the eukaryotic proteins that are involved in vesicle traffic and any bacterial or archaeal proteins.

In the course of genome annotation and analysis for the hyperthermophilic crenarchaeon *Ignicoccus hospitalis *(Podar et al, manuscript in preparation), a significant sequence similarity was found between one of the several V4R proteins encoded in the genome (Ig1332) and the Bet3 family of proteins (pfam04051), which are constituents of the eukaryotic transport protein particle complex TRAPP1. The sequence similarity was detected using HMM analyses of Pfam domains present in the *Ignicoccus *proteome and RPS-BLAST searches with the Conserved Domains Database (CDD) at NCBI. A CDD search using the Ig1332 sequence as query retrieved TRAPP_Bet3 (pfam 04051) and COG1719 (V4R domain-containing orthologs) as equally strong (E-value = 0.03) and overlapping hits.

Proteins containing the V4R domain are encoded in the sequenced genomes of numerous bacteria and archaea, with a patchy distribution and without a strict link to any specific metabolic or taxonomic markers. Described as a predicted hydrocarbon-binding domain (COG1719) [2], V4R can be present either by itself or fused with other domains, primarily involved in transcription regulation and signal transduction. Limited experimental evidence connects V4R proteins to allosteric regulation of unrelated reactions that involve hydrophobic molecules in two species of bacteria [3,4]. On the basis of these data, it is thought that V4R domains, in general, bind hydrophobic ligands and signal to effectors in *cis *or *trans*.

**Figure 1:**
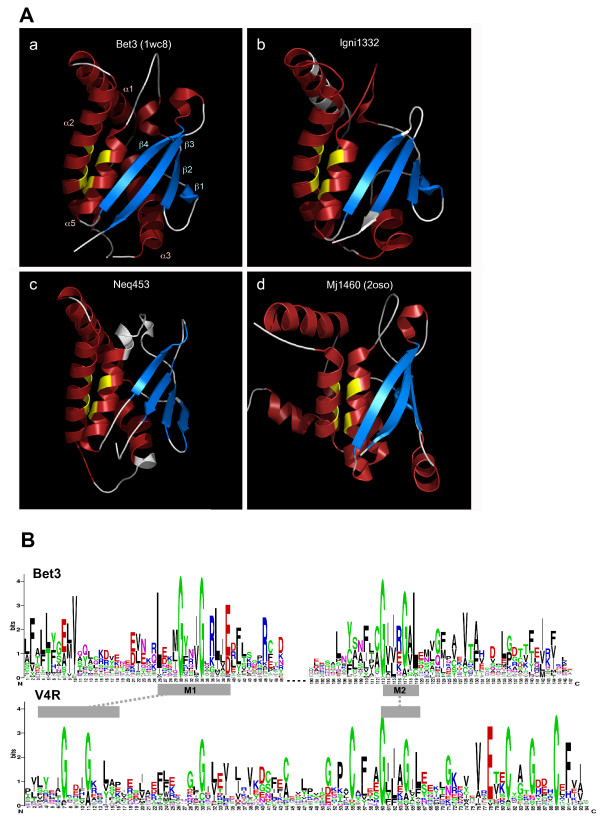
**(A) **Structure of mouse BET3 (PDB:1wc8)(a) and homology models of archaeal V4R domains (*Ignicoccus hospitalis *p1332 and *Nanoarchaeum equitans *p453)(b, c). Secondary structure inference for the archaeal proteins is overlaid on the models, coloring of the alpha helix (red) and beta strand (blue) elements corresponding to the type of prediction using PSIPRED v2.5. The location of the conserved pairs of glycines in helices 2 and 5 is indicated in yellow. The crystal structure of the *Methanocaldococcus jannaschii *p1460 (PDB, 2oso) is shown in panel d. The models were visualized using Pymol [17]. **(B) **Sequence conservation logos for eukaryotic TRAPP-Bet3 and archaeal V4R domain proteins. M1 and M2 represent previously identified motifs characteristic to the TRAPP-Bet3 family and are linked to the corresponding structure and sequence regions in V4R. The sequence logos were generated using WebLogo3 [18](the sequence alignments used as input are available upon request).

The TRAPP1 complex, which is present in all sequenced eukaryotes including basal lineages [1], is attached via the essential Bet3 subunit to the Golgi surface and captures specific endoplasmic reticulum-derived vesicles, bringing them within reach for membrane fusion [5]. The Bet3 family encompasses several different paralogs (Bet3, Tpc6/trs33, Tpc5/trs31) that, despite low sequence similarity, adopt a unique version of the α/β-pleat fold and function as homo- or heterodimers in TRAPP [6-8]. Since the primary sequence conservation within and between the two families is relatively low, we explored the potential structural conservation between V4R and Bet3 using the available high resolution crystal structures of Bet3 family proteins. Two different V4R sequences were used, *I. hospitalis *protein Igni1332 and Neq453, encoded in the genome of *Nanoarchaeum equitans*, a symbiont/parasite of *Ignicoccus *[9]. The two sequences were analyzed using the fold recognition meta-server PHYRE [10], which generated a 50–70% confidence hit to Bet3 family proteins structures. To obtain structure models of the *I. hospitalis *and *N. equitans *V4R domains, the sequences were first aligned by hand to those of TPC6 (PDB:2bjn) and BET3 (PDB:1wc8) which served as templates. Side chain residues of the corresponding template structure were replaced with those of the target V4R protein, and improper stereochemistry and atomic clashes introduced by the substitutions, insertions or deletions were then corrected and refined using the "nest" routine of the software package JACKAL [11]. Secondary structure elements were independently predicted using PSIPRED v2.5 [12] and the prediction was overlaid on the three-dimensional model with color-coding corresponding to the type of elements. As shown in Figure 1A, there is a high degree of structural similarity between Bet3 and V4R, strongly suggesting that these families of proteins are evolutionarily related. The predicted secondary structure elements are highly conserved within each family and between them, which is compatible with the presence of the same, conserved fold. A comparison with the crystal structure for a V4R protein from the archaeon *Methanocaldococcus jannaschii *(PDB, 2oso) that has been recently released by the Joint Center For Structural Genomics supports the computationally inferred model. In the VAST database of structural neighbors [13] the Bet3 and V4R structures are reciprocal best hits, with 116 residues included in the structural alignment and an E-value < 10^-5^, which is indicative of homology.

The overall architecture of Bet3 and V4R consists of five helices on one side and a twisted antiparallel four-stranded β-sheet on the other side. In the Bet3 family, two motifs, that have been previously described and are parts of helices α2 and α5, each contain a conserved pair of glycine residues separated by three amino acids (Figure 1). Based on the Bet3 and Tpc6 crystal structures, the glycine pairs allow tight packing of the α2–α5 helices at a distance incompatible with amino acids having a longer side chain. Notably, these two glycine doublets are conserved in most V4R domains as well, suggesting the existence of a similar, functionally important structural arrangement. Furthermore, around these glycines, additional conserved sequence features can be recognized between Bet3 and V4R (Figure 1B). These observations reinforce the conclusion that the Bet3 and V4R domains share a common origin and possess highly similar structural and, presumably, functional elements.

The functions of the V4R domain-containing proteins in archaea and bacteria are not thoroughly understood. Assuming that the primary role of this domain is in regulation and signaling [2], the recruitment of V4R for vesicular transport would have involved exaptation [14] during the buildup of the eukaryotic trafficking machinery at an early stage of eukaryogenesis. Alternatively, as V4R domains bind hydrophobic molecules, it cannot be ruled out that some of them are already involved in vesicular transport in prokaryotes. Vesicle formation is well known in some bacteria and archaea, although no evolutionary connection to the analogous eukaryotic process has been established [15]. Interestingly, *Ignicoccus hospitalis*, which encodes seven V4R-containing proteins (an unusually large number of compared to any other archaeon), produces vesicles that migrate through a large periplasmic space and can fuse to the outer membrane or the cytoplasmic membrane via a still uncharacterized mechanism [16]. If such a vesicle transport system exists in prokaryotes and employs the V4R domain, it could have been inherited by the LECA and subsequently underwent a major diversification at an early stage of eukaryotic evolution. Distinguishing this scenario from the exaptation hypothesis will depend on in-depth functional characterization of bacterial and archaeal V4R proteins, discovery of prokaryotic homologues to other eukaryotic vesicle transport machinery proteins, and further comparative analysis of vesicle transport systems in diverse eukaryotic lineages.

## Competing interests

The author(s) declare that they have no competing interests.

## Authors' contributions

MP identified the similarity between V4 and Bet3, contributed to sequence analysis and wrote the manuscript, MW performed the structural modeling and analysis, KSM and EVK contributed to sequence analysis. All authors read, edited and approved the final version of the manuscript.

## Reviewers' comments

Gáspár Jékely: I read the paper and find it interesting and well written. I don't have any criticism.

Mark Ragan: The Golgi complex *per se *has been secondarily modified, sometimes substantially, in parasitic basal eukaryotes such as *Giardia *and *Spironucleus *(for a comprehensive recent review, see Sokolova *et al*., *Cell and Tissue Biology *1(4):305–327, 2007). It could be interesting to explore, in somewhat more detail, the presence and interactions of the Bet3 subunit of the TRAPP1 complex in relation to the presence/absence, localisation and functions of other proteins involved in intracellular vesicle trafficking in these organisms.

My other comment relates to the discussion of a prokaryotic origin of V4R/Bet3. A broad (if patchy) distribution across prokaryotes does indeed suggest that the presence of V4R is ancestral among prokaryotes. This does not necessarily require, however, that LECA "inherited" one or more V4R homologs from a prokaryote, or that this homolog was already active in an ancient vesicle transport system in prokaryotes, as the final sentence of text indicates. The possibility is fascinating, but other options exist for moving V4R into the early eukaryotic lineage, including genome fusion and endosymbiotic transfer. Note that V4R could have been exaptive at that point within prokaryotes, as proposed for later within the eukaryote lineage.
